# Imaging alterations of cardiomyocyte cAMP microdomains in disease

**DOI:** 10.3389/fphar.2015.00172

**Published:** 2015-08-25

**Authors:** Alexander Froese, Viacheslav O. Nikolaev

**Affiliations:** ^1^Department of Cardiology and Pulmonology, Heart Research Center Göttingen, Georg August University Medical Center, Göttingen, Germany; ^2^German Center for Cardiovascular Research, University Medical Center Hamburg-Eppendorf, Hamburg, Germany; ^3^Institute of Experimental Cardiovascular Research, Hamburg, Germany

**Keywords:** cAMP, microdomain, cardiomyocyte, hypertrophy, heart failure, FRET, biosensor

## Abstract

3′,5′-cyclic adenosine monophosphate (cAMP) is an important second messenger which regulates heart function by acting in distinct subcellular microdomains. Recent years have provided deeper mechanistic insights into compartmentalized cAMP signaling and its link to cardiac disease. In this mini review, we summarize newest developments in this field achieved by cutting-edge biochemical and biophysical techniques. We further compile the data from different studies into a bigger picture of so far uncovered alterations in cardiomyocyte cAMP microdomains which occur in compensated cardiac hypertrophy and chronic heart failure. Finally, future research directions and translational perspectives are briefly discussed.

3′,5′-cyclic adenosine monophosphate (cAMP) is an important second messenger and critical regulator of cardiac function. Stimulation of cardiac contractility by catecholamines and their receptors, in particular β-adrenoceptors (β-ARs) which are central to the well-established physiological fight-or-flight response, leads to generation of cAMP that acts in distinct subcellular microdomains (Fischmeister et al., 2006; Zaccolo, 2009; Perera and Nikolaev, 2013). Such microdomains are formed around specific scaffolding proteins (i.e., A-kinase anchoring proteins or AKAPs) which create multiprotein signalosomes. They contain local pools of kinases targeted to their substrates, certain subsets of phosphodiesterases (PDEs) which are enzymes responsible for local cAMP degradation, protein phosphatases and other molecules. All of them act together to confer specificity of multiple substrate phosphorylation and therefore plethora of physiological responses engaged by the same second messenger cAMP (Buxton and Brunton, 1983; Mauban et al., 2009; Zaccolo, 2009; Diviani et al., 2011). In this mini review, we highlight most recent developments and latest research on cardiomyocyte cAMP microdomains in healthy and diseased cardiomyocytes.

## cAMP Compartmentation in Healthy Cardiomyocytes

**FIGURE 1:**
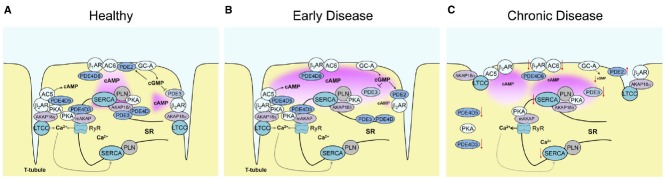
**Alterations of local cardiomyocyte cAMP signaling in early and late disease. (A)** cAMP microdomains in healthy cells formed around major calcium cycling proteins—L-type calcium channels (LTCCs), ryanodine receptor (RyR) and SERCA. Each microdomain contains local pools of PKA anchored to AKAPs and specific subsets of PDEs. β_2_-AR and AC5 are part of a signalosome around LTCC in the T-tubules controlled by PDE4D5 and PDE3 activities, while AC6 is located together with β_1_-AR at the outer membrane to produce far-reaching cAMP signals which are “channeled” to subcellular microdomains (e.g., to SERCA) by joint action of several PDEs. **(B)** Alterations of local cAMP signaling in early cardiac disease such as compensated hypertrophy. While, the total whole-cell activities of PDEs are not yet altered, there is a relocation of PDE2 between β_1_- and β_2_-AR, decrease of local PDE3 activity at β_2_-AR, and PDE3/PDE4 at SERCA. This leads to a change in cGMP/cAMP cross-talk, favoring cGMP-mediated augmentation of the contractile β_1_-AR-cAMP pool, and to disruption of the cAMP “channeling” from β_1_-AR to SERCA2a. **(C)** In chronic cardiac disease such as heart failure, multiple structural and functional alterations take place. They include partial loss of the membrane-associated T-tubules, changes in whole-cells PDE activities, delocalization of PKA, and downregulation and/or desensitization of β_1_-AR, ACs, SERCA and GC-A which all impact on receptor-microdomain coupling and functional cAMP responses.

In healthy cells, cAMP microdomains are supposed to provide specificity of A-kinase (PKA) substrate phosphorylation at different functionally relevant locations. In terms of contractility, several cAMP microdomains exist around calcium handling proteins such as L-type calcium channels (LTCCs), ryanodine receptors (RyRs), and phospholamban (PLN) which regulates the activity of the cardiac sarcoplasmic/endoplasmic reticulum (SR) calcium ATPase 2a (SERCA2a; Bers, 2002; Lompre et al., 2010). Each of these microdomains contains at least one specific AKAP and one PDE isoform (see Figure 1A). Each PDE family, e.g., PDE4, is comprised of several subfamilies such as 4A, 4B, 4D which have slightly different catalytic domain structures. Every subfamily usually has several isoforms (e.g., 4D3, 4D5) each having a unique N-terminal sequence responsible for differential subcellular localization (Conti and Beavo, 2007). Functional LTCCs are localized in cardiomyocyte transverse (T)-tubules, plasma membrane invaginations rich in caveolin (Gu et al., 2002; Insel et al., 2005). Together with AKAP15/AKAP18α (Fu et al., 2011), AKAP79 (Nichols et al., 2010) and PDE4B (Leroy et al., 2011) they form a signalosome which is crucial for β-AR/cAMP-dependent regulation of LTCC current and contractility. RyRs at the junctional SR have been claimed to be a part of mAKAP-orchestrated signalosome which also contains PDE4D3 (Lehnart et al., 2005). PLN forms a complex with AKAP18δ (Lygren et al., 2007) and one of the PDE4D and PDE3A isoforms (Beca et al., 2011, 2013) to regulate diastolic calcium uptake. Each of these complexes should also include a local pool of type II PKA molecules. There are two types of regulatory PKA subunits RI and RII, together with catalytic subunits they form either PKA type I or type II complexes. While PKA type II has been shown to phosphorylate the above mentioned calcium handling proteins, the exact nature of PKA type I substrates remains unclear (Stangherlin et al., 2011). Apart from channel recordings and substrate phosphorylation analysis, which provide only indirect measure of the downstream PKA-mediated signaling, it has been challenging to directly visualize cAMP levels in these specific microdomains until novel biophysical techniques became available.

Advent of fluorescence resonance energy transfer (FRET) based biosensors enabled a real-time monitoring of cAMP in intact cells (Sprenger and Nikolaev, 2013). Very early experiments in neonatal cardiomyocytes could directly visualize discrete microdomains where cAMP increases and activates PKA upon β-AR stimulation (Zaccolo and Pozzan, 2002). There are even different pools of cAMP associated with type I and type II PKA responsible for phosphorylation of different substrates and oppositely regulated by cGMP due to its action on either cGMP-activated PDE2 or cGMP-inhibited PDE3 (Stangherlin et al., 2011). Development of further biosensors, adenoviruses and transgenic mice which serve as a way to express such sensors in cells and tissues, enabled measurements in adult myocytes which revealed tight regulation of cAMP by various PDE families (Warrier et al., 2005; Leroy et al., 2008) and spatial differences between β_1_- and β_2_-AR-cAMP signals, the former having more diffuse far-reaching and the latter highly confined nature (Nikolaev et al., 2006).

Even deeper insights into cAMP compartmentation in relation to membrane structure of living cardiomyocytes were provided by scanning ion conductance microscopy (SICM) combined with FRET. SICM is a non-optical imaging technique which utilizes an electrolyte-filled glass nanopipette as a scanning probe fixed on a three-axis piezo-actuator stage (Korchev et al., 1997). The current flow through pipette is decreased whenever it approaches cell membrane, and by keeping this current change and thereby the distance between pipette tip and cell membrane constant, one can scan the morphological profile of the membrane with nanometer resolution. In general, SICM as a multimodal imaging technique can be applied to study not only cell/tissue structure, but also to record ion channel currents in precise membrane locations, to analyze cell volume and contractility (Miragoli et al., 2011). Using combination of SICM with FRET, which allows local nanopipette-based receptor stimulation and concomitant cAMP imaging, it was uncovered that β_1_-AR is localized across the whole membrane, while, in contrast, β_2_-AR is located exclusively in the T-tubules of healthy cells (Nikolaev et al., 2010). Moreover, β_2_AR are strictly compartmentalized in caveolin3-rich microdomains (Wright et al., 2014) to produce confined cAMP signals limited by local PKA and PDE4 activities. This receptor can also switch from stimulatory to inhibitory G-proteins to limit cAMP production upon prolonged exposure to high agonist concentrations (Liu et al., 2009).

Not only AKAPs but also cAMP synthetizing enzymes adenylyl cyclases (ACs), with the most predominantly expressed cardiac AC5 and AC6, can center cAMP signalosomes in myocytes and other cells (Cooper and Tabbasum, 2014). A recent elegant electrophysiological study by Timofeyev et al. (2013) using AC5 and AC6 knockout myocytes revealed that AC5 is mainly localized in T-tubules where it interacts with caveolin and β_2_-ARs. Together with a local PDE, caveolin is thereby involved in compartmentation of β_2_-AR-cAMP signals in this microdomain. In contrast, AC6 associated the β_1_-AR is localized outside the T-tubules and is responsible for β_1_-AR-mediated augmentation of LTCC current. Interestingly, yet another functional population of β_1_-ARs is targeted to T-tubules and the AC5-PDE signalosome (Timofeyev et al., 2013).

To gain more specific insights into cAMP dynamics directly in the microdomains around calcium handling proteins, our group has recently generated targeted FRET biosensors and expressed them in myocardium of transgenic mice to directly monitor local cAMP in freshly isolated adult cardiomyocytes. First, the cAMP sensor Epac1-camps was targeted to caveolin-rich membrane microdomains (to generate pmEpac1-camps) where it should localize in close proximity to LTCC and β_2_-AR (Perera et al., 2015). This new sensor uncovered differential PDE-dependent regulation of β_2_- and β_1_-AR stimulated cAMP pools at the membrane, the former one predominantly confined by PDE3 and the latter one by balanced actions of PDE4, 3, and 2 (Perera et al., 2015). Second, a fusion of Epac1-camps with PLN was used to target the cAMP sensor to the SERCA2a microdomain (Sprenger et al., 2015). Here, high basal PDE3 and PDE4 effects were detected which confine this microdomain and prevent PLN phosphorylation by high cytosolic cAMP levels. More interestingly, upon β-adrenergic stimulation, local, and cytosolic PDE3 and PDE4 act in concert to “channel” cAMP from the membrane to SERCA2a and enable functional response in this microdomain (Sprenger et al., 2015; Figure 1A), a phenomenon which has previously been observed in HEK293 cells when measuring membrane, cytosolic and nuclear cAMP pools (Terrin et al., 2006). However, particularly exiting findings could be made when subjecting both FRET sensor transgenic mouse lines to an experimental model of cardiac disease.

## Remodeling of cAMP Microdomains in Early Cardiac Disease

While alterations in cAMP pathway have been extensively studied in chronic disease (see below), not much is known about changes in local cAMP signaling in early compensated cardiac hypertrophy. To address this question, pmEpac1-camps and Epac1-camps-PLN mice were subjected to transverse aortic constriction which induces only a mild compensated phenotype in the FVB/N1 mouse background. Interestingly, in this case, no changes in total whole-cell PDE activities and no β_1_-AR or guanylyl cyclase A (GC-A, membrane receptor which produces cGMP upon natriuretic peptide stimulation) desensitization can be detected. Instead, there was a subcellular relocation of PDE2 between β_1_- and β_2_-AR, and local decrease of the major PDE3-mediated control at the β_2_-AR (Figure 1B). This leads to a change of cGMP/cAMP cross-talk in a way that cGMP, which is produced by GC-A stimulated with increased levels of natriuretic peptides in hypertrophy, leads to augmentation of the far-reaching β_1_-AR-cAMP pools coupled to increase in force and frequency of contraction (Perera et al., 2015). This might represent a compensatory mechanism aimed to initially maintain cardiac output under the conditions of increased pressure overload during disease, before the transition to a decompensation at some later time-point. However, the exact local mechanisms which accompany this transition remain to be defined. It is also not clear in which particular membrane microdomains GC-A is localized and whether this localization is changed in disease.

The study using cAMP biosensor targeted to SERCA2a demonstrated that cardiac hypertrophy leads to local decrease of PDE3 and PDE4 effects which confine this microdomain from the bulk cytosol. Furthermore, it causes changes in PDE composition at various subcellular locations in a way that leads to impairment of the above described PDE3/4-dependent “channeling” of cAMP from β_1_-AR to SERCA2a (Figure 1B, Sprenger et al., 2015). In the future, it would be exciting to dissect which individual PDE3 and PDE4 isoforms are involved in any individual microdomain, how they are regulated by calcium signaling and positive or negative feedback loops, and how all these processes are affected by cardiac disease. This can be done using PDE knockout mouse models, as previously demonstrated for healthy neonatal mouse myocytes using pmEpac-camps and its parential cytosolic sensor (Leroy et al., 2011; Mika et al., 2015). Future developments should also provide new biosensors for other microdomains, such as the one associated with RyR, various other signalosomes and organelles.

## cAMP Microdomain Alteration in Chronic Cardiac Disease

In human and rodent failing myocytes, a series of well-established signaling alterations occurs, including desensitization/downregulation of β_1_-AR, GC-A, ACs, SERCA2a, and impairment of PKA-dependent phosphorylation of major contractile substrates (Lohse et al., 2003). Structurally, SICM studies in failing human and rat cardiomyocytes revealed a loss of cell-surface T-tubules as well as disruption of Z-groove structure (Lyon et al., 2009). The whole-cell activities of major PDE families were reported to be down- (for PDE3/4; Ding et al., 2005; Abi-Gerges et al., 2009) or upregulated (PDE2; Mehel et al., 2013). Loss of membrane T-tubules leads to redistribution of β_2_-AR to detubulated areas where it gets uncoupled from its microdomain and generates far-reaching cAMP signals (Nikolaev et al., 2010; see Figure 1C). Altered cAMP compartmentation worsens PKA substrate phosphorylation and calcium cycling. Several open question still remain. Is there any PDE relocation also in chronic disease? Are there any differences between various clinical types of heart failure and what is the time course of deleterious events (detubulation, receptor relocation, microdomain remodeling) during progression of disease? Still unclear are the exact molecular mechanisms behind the loss of T-tubules and their link to calcium cycling, improvement of which correlates with restoration of the membrane structure (Lyon et al., 2012).

Better understanding of cAMP dynamics in various functionally relevant microdomains and especially of their changes in disease should ultimately provide more precise ways of therapeutic correction. To improve cAMP or cGMP flow in the microdomains, one can imagine approaches aimed at depletion of specific PDEs or PKA from desired signalosomes. More specific PDE inhibition and treatments aimed at improvement of membrane structure, receptor localization and protein composition of the microdomains can also be considered. These developments should enable more targeted and specific cardiac therapeutics.

## Author Contributions

AF and VN discussed the concept, wrote and edited the manuscript.

### Conflict of Interest Statement

The authors declare that the research was conducted in the absence of any commercial or financial relationships that could be construed as a potential conflict of interest.
